# Optimizing hospital distribution across districts to reduce tuberculosis fatalities

**DOI:** 10.1038/s41598-020-65337-x

**Published:** 2020-05-25

**Authors:** Mi Jin Lee, Kanghun Kim, Junik Son, Deok-Sun Lee

**Affiliations:** 1 Department of Physics, Inha University, Incheon, 22212 Korea; 2 Financial Engineering Team, Meritz Securities, Seoul, 07326 Korea; 3 Department of Family Medicine, Daejeon Sun Medical Center, Daejeon, 34811 Korea

**Keywords:** Statistical physics, Epidemiology, Scientific data

## Abstract

The spatial distributions of diverse facilities are often understood in terms of the optimization of the commute distance or the economic profit. Incorporating more general objective functions into such optimization framework may be useful, helping the policy decisions to meet various social and economic demands. As an example, we consider how hospitals should be distributed to minimize the total fatalities of tuberculosis (TB). The empirical data of Korea shows that the fatality rate of TB in a district decreases with the areal density of hospitals, implying their correlation and the possibility of reducing the nationwide fatalities by adjusting the hospital distribution across districts. Approximating the fatality rate by the probability of a patient not to visit a hospital in her/his residential district for the duration period of TB and evaluating the latter probability in the random-walk framework, we obtain the fatality rate as an exponential function of the hospital density with a characteristic constant related to each district’s effective lattice constant estimable empirically. This leads us to the optimal hospital distribution which finds the hospital density in a district to be a logarithmic function of the rescaled patient density. The total fatalities is reduced by 13% with this optimum. The current hospital density deviates from the optimized one in different manners from district to district, which is analyzed in the proposed model framework. The assumptions and limitations of our study are also discussed.

## Introduction

Complex systems are organized, by evolution or design, to satisfy the optimization conditions including the minimization of the traveling time in the transportation system^1^ and the maximization of the stability of the airline networks^2^, the resilience of the power-grid system^3,4^, and the growth rate of cellular networks^5^. Likewise, the locations of facilities are expected to be subject to various optimization conditions^6–8^. Despite the complexity of the facility location decisions^9,10^, the empirically observed distributions of facilities often show simple and universal features, revealing the nature of the underlying optimization problem. Most remarkably, the spatial density of facilities scaling with the population density^11–13^ with exponent 2/3 or $1$ implies that they are distributed to minimize the social opportunity cost such as the commute distance or to maximize the economic profit depending on the distribution of available customers^14^.

For coping with diverse social or economic demands in real-world applications, the objective function in the facility distribution optimization may need to be expanded beyond the commute distance or profit. Towards developing such a general theory, here we consider as an example the problem of distributing hospitals across districts to minimize the total fatalities of tuberculosis (TB) by using the empirical data of Korea. While the chemotherapy for TB is well established, showing a success rate as high as 85% on average^15^, TB spreads annually to about 10 million patients, being a major cause of death worldwide^15,16^. In Korea, the incidence of TB is 77 per 100000 as of 2016, which is high compared with other developed countries, e.g., the member countries of the Organization for Economic Cooperation and Development^17^. Patients with TB can be cured if they are diagnosed and get treatment timely. Visiting a hospital and taking drugs for about 6 months are necessary for the full recovery from TB^18^, which may not be easy from the patients’ perspective. Therefore, the accessibility of local hospitals and the well-trained attending staff providing consistent treatment and care should be crucial for the treatment of TB^16,19^, which is recognized also in the reports of the World Health Organization^15^. The correlation between the hospital distribution and the fatality rate of TB in a district is indeed identified in the Korea TB data-sets which we will analyze in the present study; The fatality rate in a district tends to decrease as the areal density of hospitals therein increases, which is an important point demanding a quantitative explanation and leads us to expect that relocating hospitals across districts may reduce the total fatalities of TB nationwide.

The optimal distribution of hospitals across districts minimizing the total TB fatalities depends on the concrete form of the fatality rate as a function of the hospital density, which is, however, unknown; The empirically observed negative dependence cannot give this information, as districts are different not only in the hospital density but also in various other properties such as area or population. To address the district-dependent fatality rate, we take a modeling approach, in which the fatality rate is assumed to be identical to the probability of a patient *not* to visit a hospital and get the medical treatment for the duration period of TB. This is motivated by the expectation that a patient is very likely to be cured once she/he gets a proper treatment in a hospital, given the high success rate of the TB chemotherapy equally applicable to all districts. In this framework, the fatality rate turns out to be an exponentially decaying function of the hospital density, and we are able to derive the optimal hospital densities in all districts the collection of which decreases the total fatalities of TB by 13% from the current value. The predicted optimal hospital density is given by a logarithmic function of the rescaled patient density. Our results delineate an analytic approach to the facility optimization problem under an objective function from a public health perspective. The limitation and further generalization of the results will be discussed.

## Results

### TB fatality rate and hospital density: Empirical data

The incidence and mortality of TB are well recorded in Korea. In Statistics Korea^20^, we obtain for district $i=1,2,\ldots ,I=228$ in year 2014 the number of the newly reported TB patients ${N}_{i}$, the number of dead TB patients (fatalities) ${D}_{i}$, the number of private general hospitals ${H}_{i}$, and the area ${a}_{i}$. Here “district” includes three distinct units for administrative division, Gu, Gun, and Si, with the population ranging from ${10}^{4}$ to ${10}^{6}$ and smaller than the metropolitan cities.

We are interested in the fatality rate ${\phi }_{i}$ of TB, defined as the ratio of the number of dead TB patients to the number of new TB patients reported for one year in each district $i$,

1 $${\phi }_{i}\equiv \frac{{D}_{i}}{{N}_{i}}.$$

**Figure 1:**
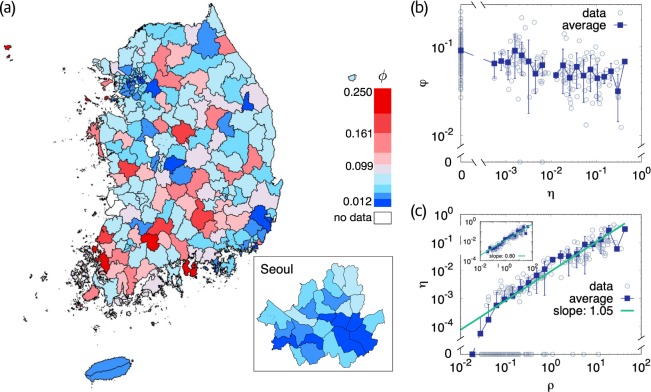
Distribution and relations of the TB fatality rate $\phi$, the areal density of hospitals $\eta$, and the patient density $\rho$ in Korean districts at the level of Gu, Gun, and Si. The unit of $\eta$ and $\rho$ is km^−2^. (**a**) The TB fatality rate $\phi$ is represented by color in 228 districts. Seoul, the capital city, has 25 Gu’s and is shown separately. (**b**) Fatality rate $\phi$ versus hospital density $\eta$. Open circles are the raw data for all districts and filled squares represent the average fatality rate for each given hospital density with the standard deviations as errorbars. The Pearson correlation coefficient is −0.26 with P-value 0.000070 for all districts and −0.25 with ${\rm{P}}=0.0025$ for the districts with both $\eta$ and $\phi$ non-zero. (**c**) Hospital density $\eta$ versus patient density $\rho$. As in (**b**), open circles and filled squares represent the raw data and the average value, respectively. The solid line fits the averaged data and its slope is $1.05\pm 0.07$. Inset: The same plot for the districts with $\eta  > 0$. The fitting line has slope $0.80\pm 0.045$.

It is quite different from district to district, ranging between $0.01$ and $0.25$, as shown graphically in Fig. 1(a). What drives such difference in the TB fatality rate? Taking regularly medical treatments and examinations in hospitals may be the most important for curing TB, which is available in the easy-to-frequently-access medical environment established in the local community. Therefore a difference in the abundance and accessibility of hospitals in the patients’ residential districts will be a major factor giving rise to such variation of the fatality rate with district. In this light, we investigate the relation between the fatality rate ${\phi }_{i}$ and the areal hospital density

2 $${\eta }_{i}\equiv \frac{{H}_{i}}{{A}_{i}},$$

in unit of km^−2^. In Fig. 1(b) ${\phi }_{i}$ tends to decrease with ${\eta }_{i}$; The larger the hospital density is, the smaller the fatality rate is. This correlation is significant with ${\rm{P}} < {10}^{-4}$. Yet the dependence does not look so strong as expected. This will be shown to be due to that the fatality rate of a district may depend not only on the hospital density but also on other characteristics. In this work, all correlation values are measured in linear scales.

What principle underlies the current spatial distribution of hospitals? The scaling behavior with respect to the patient density has hinted at the answer^14^. The hospital density scales with the areal TB patient density ${\rho }_{i}\equiv \frac{{N}_{i}}{{A}_{i}}$ as

3 $${\eta }_{i}\sim {\rho }_{i}^{\alpha },$$

in which $\alpha =1.05\pm 0.07$ when all districts are included, and $\alpha =0.80\pm 0.045$ when the districts having no private general hospital are excluded [Fig. 1(c)]. Many other properties also scale with respect to the patient density. The patient density is almost linearly related to the population density ${\rho {\prime} }_{i}={P}_{i}/{A}_{i}$ with ${P}_{i}$ the number of people living in district $i$ [Fig. S1]. The exponent $\alpha$ for the hospitals in United States is close to $1$, rather than 2/3^14^. These results suggest that the profit maximization affects the hospital distribution. For self-containment, let us sketch the corresponding optimization calculations. The sum of the economic profits of all hospitals distributed across *I*′ districts in a country is given by

4 $${E}_{{\rm{profit}}}=\mathop{\sum }\limits_{i=1}^{I{\prime} }\,{H}_{i}\,\omega \left(\frac{{N}_{i}}{{H}_{i}}\right)=\sum _{i}\,{A}_{i}\,{\eta }_{i}\,\omega \left(\frac{{\rho }_{i}}{{\eta }_{i}}\right)$$

with $\omega (x)$ the expected profit of a single hospital having $x$ patients available. On the other hand, the sum of the social costs, such as the travel distances, of patients is given by

5 $${E}_{{\rm{cost}}}=\mathop{\sum }\limits_{i=1}^{I{\prime} }\,{N}_{i}\,\psi \left(\frac{{A}_{i}}{{H}_{i}}\right)=\sum _{i}\,{A}_{i}\,{\rho }_{i}\,\psi \left(\frac{1}{{\eta }_{i}}\right)$$

with $\psi (x)={x}^{1/2}$ being the expected travel distance of a patient residing in a district of $x$ area per hospital. Then, for a fixed total number of hospitals

6 $${H}_{{\rm{total}}}=\mathop{\sum }\limits_{i=1}^{I{\prime} }\,{H}_{i}=\sum _{I}\,{A}_{i}{\eta }_{i},$$

one finds, by solving $\frac{\partial {E}_{{\rm{profit}}}}{\partial {\eta }_{i}}=0$, *E*_profit_ to be maximized when $\frac{{\rho }_{i}}{{\eta }_{i}}={\rm{const}}.$, corresponding to $\alpha =1$, and by solving $\frac{\partial {E}_{{\rm{cost}}}}{\partial {\eta }_{i}}=0$, ${E}_{{\rm{c}}ost}$ to be minimized when $\frac{{\rho }_{i}}{{\eta }_{i}^{3/2}}={\rm{const}}.$, corresponding to $\alpha =2/3$^14^.

Our question is then whether the current hospital distribution, seemingly maximizing the economic profit, is the best also for minimizing the total fatalities of TB

7 $${E}_{{\rm{fatalities}}}=\mathop{\sum }\limits_{i=1}^{I{\prime} }\,{D}_{i}=\sum _{i}\,{N}_{i}\,{\phi }_{i}.$$

Can ${E}_{{\rm{fatalities}}}$ be reduced by the redistribution of hospitals across district, i.e., some change of $\{{\eta }_{i}\}$? To answer this, we should formulate the total fatalities in Eq. (7) as the objective function and minimize it with respect to the hospital density for the given total number of hospitals in Eq. (6). The fatality rate ${\phi }_{i}$ should be some function of the hospital density ${\eta }_{i}$. If the optimal hospital densities $\{{\eta }_{i}^{({\rm{opt}})}\}$ are obtained by this optimization computation, we will be able to evaluate the quality of the current spatial distribution of hospitals regarding its capacity of TB treatment. Also we will see immediately how to redistribute the hospitals to reduce the TB fatalities. In the present study we do not consider a variation in the numbers of TB patients $\{{N}_{i}\}$ but take them for given; The onset and spreading of the TB or a general epidemic disease depend strongly on the topology of human contact networks and the infection rate, which is another important research topic and has been studied extensively^21,22^.

### Fatality rate as a function of hospital density: Model

The empirical fatality rate ${\phi }_{i}$ in Eq. (1) can be considered as the probability of a TB patient to die, losing the opportunity to get proper medical treatment in time. Our idea is to approximate the latter by the probability that a patient does not visit any hospital in her/his residential district for a given period ${t}_{({\rm{TB}})}=3$ years, the empirically reported period of TB duration from onset to either cure or death^23^. In this model framework, it determines the fate of a TB patient whether she/he visits a hospital or not for the period of ${t}_{({\rm{TB}})}$. The patient will recover if yes, but will be dead otherwise. One can see that this is a trapping problem^24^ from the viewpoint of a patient; Once a patient (walker) reaches a hospital (trap), she loses the status of a patient (absorbed at the trap). The probability of a walker to *survive* during a given number of steps corresponding to ${t}_{({\rm{TB}})}$ in this trapping problem is translated into the *fatality* rate of a TB patient in reality.

Suppose that $H$ traps are uniformly and independently distributed in a two-dimensional Euclidean lattice of $L\times L$ sites and that a walker walks around the region, who disappears on reaching any one of the traps. Then the probability of the walker to survive (not to reach any of the traps) after $\tau$ steps is given by

8 $$\phi =\langle {(1-\lambda )}^{S(\tau )}\rangle ,$$

where $\lambda =\frac{H}{{L}^{2}}$ is the density of traps and $S(\tau )$ is the number of distinct sites visited up to $\tau$ steps. $\langle \cdots \rangle$ represents the average over different realizations of walks. In the limit $|\log (1-\lambda )\frac{{\sigma }_{S(\tau )}^{2}}{\langle S(\tau )\rangle }|\ll 1$ reachable when the trap density is sufficiently low or the number of steps is small enough, the survival probability $\phi$ can be approximated in terms of the first cumulant of the probability distribution of $S$ as^25^

9 $$\phi ={e}^{-\lambda \langle S(\tau )\rangle },$$

which is the exponential function of the trap density $\lambda$. It seems that Eq. (9) allows us to relate the hospital density and the fatality rate. However the dimensionless quantities $\lambda$ and $\langle S(\tau )\rangle$ are not directly available. In random walks in two dimensions, the expected number of distinct visited sites is known to be^24^

10 $$\langle S(\tau )\rangle \propto \frac{\tau }{\log \,\tau },$$

which is inserted into Eq. (9) to give

11 $$\phi =\exp \,\left(-\,c\frac{\tau }{\log \,\tau }\lambda \right)$$

with the coefficient $c=3.5/{1.13}^{2}$ known numerically^26^. In the opposite limit $|\log (1-\lambda )\frac{{\sigma }_{S(\tau )}^{2}}{\langle S(\tau )\rangle }|\gg 1$, the survival of the walker is governed by the probability of a large trap-free region to be formed, which leads to a stretched exponential form $\log \,\phi \sim \sqrt{\lambda \tau }$^26–28^.

The exponential decay of $\phi$ with $\lambda$ in Eq. (9) holds when the hospital density is sufficiently low. The randomness of the mobility pattern is assumed in obtaining Eq. (11). We should remark that the human mobility pattern revealed by tracing the travel routes of bank notes^29^ or the mobile phone records^30^ displays deviation from random walk; The radius of gyration of individual trajectories grows logarithmically with time^30^, in contrast to the square-root scaling in the conventional random walk, and such slow diffusion is known to arise under the memory effect^31–33^ or the spatial quenched disorder^24^. The assumption we make about the human mobility pattern is that the *coarse-grained* trajectories of individuals on the time scale of ${t}_{({\rm{TB}})}=3$ years, much longer than the previous studies, show the survival probability given in Eq. (11) like random walks. The coarse-grained trajectory is obtained by neglecting the spots swiftly passed by and connecting the remaining notable places which an individual visits and stays for a while in, such as her/his house, workplace, parks, stores, banks, oil stations, and hospitals. In our model, we are interested in whether a hospital is included in the list of such notable places. We cannot check directly the validity of Eqs. (9) and (11), however, we will present indirect evidence that they are reasonable assumptions.

### Lattice constant and dimensionless quantities

To relate the survival probability in the 2D trapping problem to the fatality rate of TB, we need to convert the empirical data into dimensionless ones of Eq. (11). To this end, we discretize the region of each district $i$ by introducing the lattice constant ${a}_{i}$, corresponding to the typical length of one single step or the average distance between adjacent notable places appearing in the coarse-grained trajectories. Then the district is represented by the ${L}_{i}\times {L}_{i}$ Euclidean lattice with ${L}_{i}=\sqrt{\frac{{A}_{i}}{{a}_{i}^{2}}}$, for which the areal hospital density ${\eta }_{i}={H}_{i}/{A}_{i}$ is converted to the dimensionless hospital density ${\lambda }_{i}$ as

12 $${\lambda }_{i}=\frac{{H}_{i}}{{L}_{i}^{2}}=\frac{{H}_{i}}{\frac{{A}_{i}}{{a}_{i}^{2}}}={a}_{i}^{2}{\eta }_{i}.$$

Let ${\ell }_{({\rm{TB}})}$ be the typical travel distance of an individual for ${t}_{({\rm{TB}})}=3$ years. Then the number of steps taken in her/his coarse-grained trajectory for ${t}_{({\rm{TB}})}$ in a district $i$ will be given by

13 $${\tau }_{i}=\frac{{\ell }_{({\rm{TB}})}}{{a}_{i}}.$$

Plugging Eqs. (12) and (13) into Eq. (11), we find the fatality rate represented as

14 $${\phi }_{i}=\exp \,\left(\,-\,\frac{{\eta }_{i}}{{\tilde{\eta }}_{i}}\right)$$

with the characteristic hospital density ${\tilde{\eta }}_{i}$ given by

15 $${\tilde{\eta }}_{i}={\left(c\frac{{\tau }_{i}}{\log {\tau }_{i}}{a}_{i}^{2}\right)}^{-1}={\left(c\frac{{\ell }_{({\rm{TB}})}{a}_{i}}{\log \left(\frac{{\ell }_{({\rm{TB}})}}{{a}_{i}}\right)}\right)}^{-1}.$$

Assuming the validity of Eq. (14), one can estimate the characteristic hospital density ${\tilde{\eta }}_{i}$ by using the empirical data of the fatality rate ${\phi }_{i}$ and the hospital density ${\eta }_{i}$ in Eq. (14) as

16 $${\tilde{\eta }}_{i}=\frac{{\eta }_{i}}{|\log \,{\phi }_{i}|}.$$

**Figure 2:**
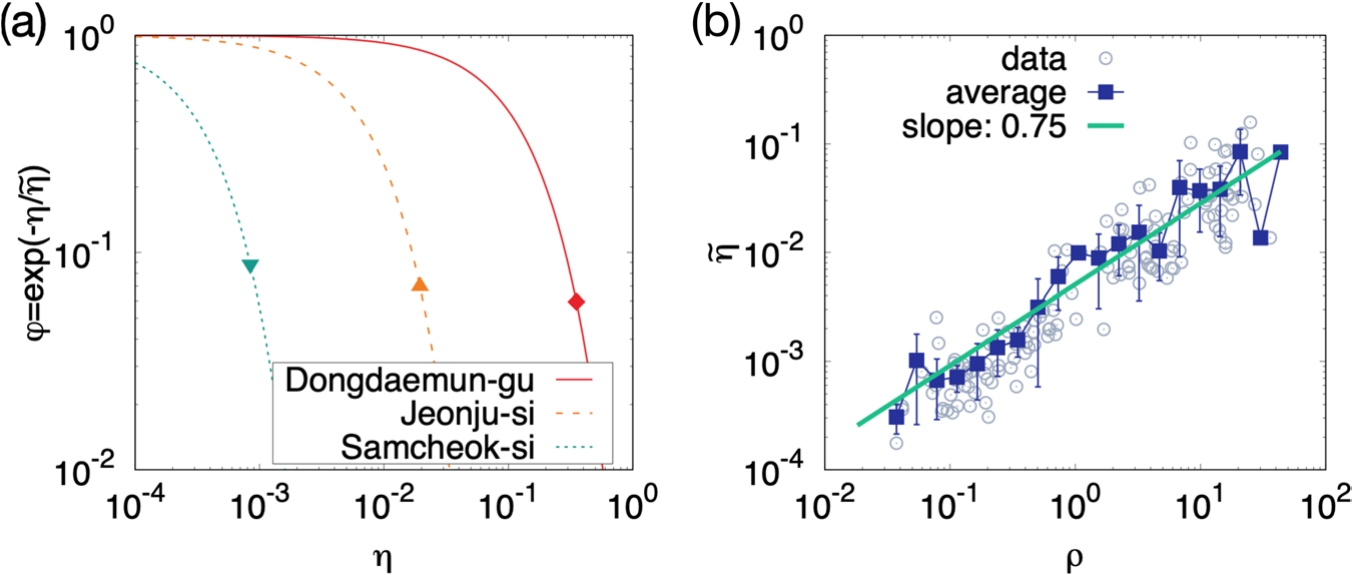
Theoretical prediction for the fatality rate and the estimated characteristic hospital density. (**a**) The theoretical prediction, Eq. (14), for the fatality rate $\phi$ as a function of the hospital density $\eta$ for selected districts having $\tilde{\eta }=1.2\times {10}^{-1}$, $7.3\times {10}^{-3}$, and $3.5\times {10}^{-4}\,{{\rm{km}}}^{-2}$, respectively. Filled points represent the real data for each district. (**b**) Plot of $\tilde{\eta }$ versus the patient density $\rho$. Open circles and filled squares indicate the real data and the average, respectively. The solid line fits the average of $\tilde{\eta }$ as a function of $\rho$ and the slope is $0.75\pm 0.056$.

The exponential functions ${\phi }_{i}(\eta )$’s in Eq. (14) with the estimated coefficient ${\tilde{\eta }}_{i}$ for selected districts are shown in Fig. 2(a). ${\tilde{\eta }}_{i}$ is different from district to district, growing with the patient density [Fig. 2(b)], which underlies the weaker decay of the fatality rate with the hospital density [Fig. 1(b)] than would be expected if ${\tilde{\eta }}_{i}$ were identical for all districts. The estimated ${\tilde{\eta }}_{i}$ is the characteristic constant of each district and will be used throughout the optimization computation.

**Figure 3:**
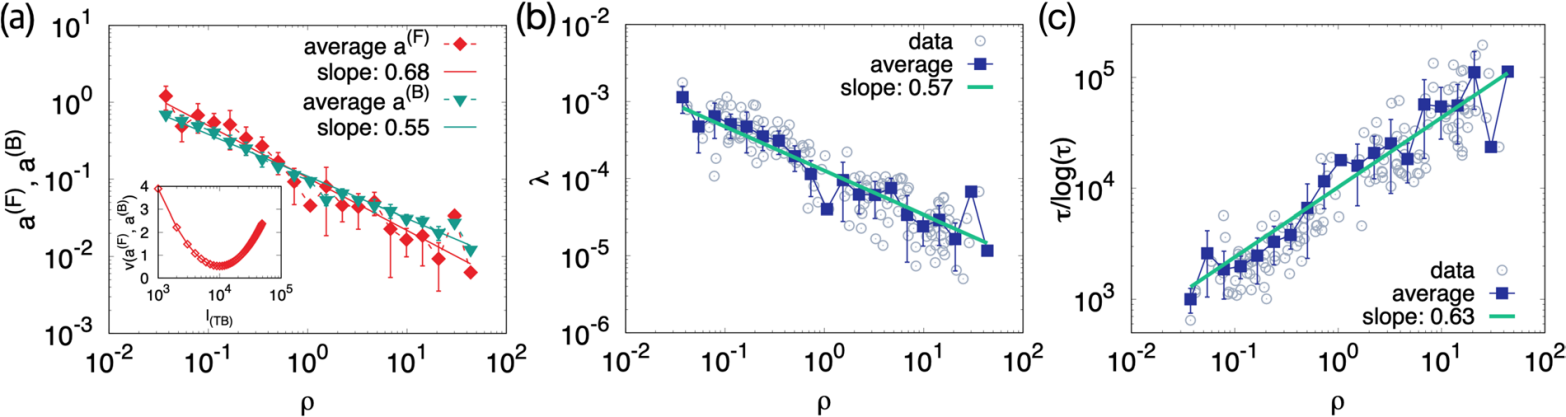
Lattice constant and dimensionless quantities. (**a**) Lattice constants ${a}^{({\rm{F}})}={a}^{({\rm{F}})}({{\ell }_{({\rm{TB}})}}^{\ast })$ and ${a}^{({\rm{B}})}$ as functions of the patient density $\rho$ in logarithmic scales. The errorbars are standard deviations. The fitting lines have slopes $-\,0.68\pm 0.044$ and $-\,0.55\pm 0.019$, respectively. Inset: The average logarithmic distance $v$ is minimized at ${\ell }_{({\rm{TB}})}^{\ast }=10000$ km with errorbar 1000 km. (**b**) Dimensionless hospital density $\lambda =\eta {a}^{2}$ [Eq. (12)] versus patient density. The dashed line with filled squares represents the average values with the errorbars being standard deviations. The solid line fits the average values and has slope $-\,0.57\pm 0.050$. (**c**) Plot of $\frac{\tau }{\log \,\tau }$ versus patient density. The dashed line with filled squares and errorbars represent the average values and standard deviations. The slope of the solid line is $0.63\pm 0.046$.

The lattice constant can be obtained by using the estimated ${\tilde{\eta }}_{i}$ in Eq. (15) and solving for ${a}_{i}$. Let us denote the solution by ${a}_{i}^{({\rm{F}})}({\ell }_{({\rm{TB}})})$. To validate it, we compare it with another estimate independent of the empirical values of the fatality rate or the hospital density. We use the data of the number of business buildings ${B}_{i}$ in each district^20^. The business buildings, including hospitals, are the candidates for the notable places included in the coarse-grained trajectories. The typical distance between adjacent business buildings can be a candidate for the lattice constant, which is given by

17 $${a}_{i}^{({\rm{B}})}=\sqrt{\frac{{A}_{i}}{{B}_{i}}}$$

under the assumption that the business buildings are uniformly distributed in each district. For the comparison of ${a}^{({\rm{F}})}({\ell }_{({\rm{TB}})})$ and ${a}_{i}^{({\rm{B}})}$, we take the value of ${\ell }_{({\rm{TB}})}$ minimizing the average logarithmic distance $v({a}^{({\rm{F}})},{a}^{({\rm{B}})})={\sum }_{i}\,{(\log {a}_{i}^{({\rm{F}})}-\log {a}_{i}^{({\rm{B}})})}^{2}/{\sum }_{i}\,1$, which is ${\ell }_{({\rm{TB}})}^{\ast }=10000\pm 1000$ [Fig. 3(a)]. It corresponds to the annual traveling distance 3300 km which is reasonably close to the empirical value $8478$ km of Korea^34^. In Fig. 3(a), the two lattice constants ${a}^{({\rm{F}})}={a}^{({\rm{F}})}({\ell }_{({\rm{TB}})}^{\ast })$ and ${a}^{({\rm{B}})}$ show good agreement in their magnitudes, supporting the validity of the assumptions of our model and its formulas, Eqs. (14) and (15). Due to this agreement and Eq. (17), we can see that a large or small value of ${a}_{i}^{({\rm{F}})}$ originates from the sparse or dense business buildings in district $i$.

With ${a}_{i}^{({\rm{F}})}$, the dimensionless hospital density ${\lambda }_{i}$ and the number of steps ${\tau }_{i}$ taken for ${t}_{({\rm{TB}})}$ can be evaluated by Eqs. (12) and (13), which are plotted versus the patient population density in Fig. 3(b,c), respectively. In contrast to the real hospital density ${\eta }_{i}$, ${\lambda }_{i}$ is lower in a district with higher patient density [Fig. 3(b)]. It is attributed to the smaller lattice constants in the districts of higher patient densities, arising from the denser buildings. On the other hand, the number of steps taken for ${t}_{({\rm{TB}})}$ increases as the patient density increases, increasing the chance to visit hospitals. To sum up, effectively less hospitals are distributed but the patients take more steps for the given period ${t}_{({\rm{TB}})}$ in the higher-populated districts, which explains the slightly lower fatality rates therein than in lower-populated districts as shown in Fig. 1(b).

### Optimal hospital density

The fatality rate formula in Eq. (14) is applicable to ${I}_{s}$ districts having non-zero $\eta$ and $\phi$ in the empirical data. Then one can minimize the total fatalities in those ${I}_{s}$ districts

18 $${E}_{{\rm{fatalities}}}=\mathop{\sum }\limits_{i=1}^{{I}_{s}}\,{N}_{i}{\phi }_{i}=\sum _{i}\,{A}_{i}\,{\rho }_{i}\,\exp \,\left(-\,\frac{{\eta }_{i}}{{\tilde{\eta }}_{i}}\right),$$

with respect to the hospital density distribution $\{{\eta }_{i}\}$ for fixed ${N}_{i}$, ${\tilde{\eta }}_{i}$, and total number of hospitals ${H}_{{\rm{total}}}$. In the data of year 2014, ${I}_{s}=143$, ${H}_{{\rm{total}}}=328$, and ${E}_{{\rm{fatalities}}}=1718$^20^. We allow ${H}_{i}$’s to be arbitrary real numbers, and the case of integer ${H}_{i}$’s will be discussed later. ${E}_{{\rm{fatalities}}}$ in Eq. (18) is minimized when $\delta E={\sum }_{i}\,{A}_{i}\delta {\eta }_{i}\left(-\,\frac{{\rho }_{i}}{{\tilde{\eta }}_{i}}{e}^{\frac{-{\eta }_{i}}{{\tilde{\eta }}_{i}}}+z\right)=0$ and ${\partial }^{2}{E}_{{\rm{fatalities}}}/\partial {\eta }_{i}^{2} > 0$ with $z$ being the Lagrange multiplier. Consequently the optimal hospital density is found to be

19 $${\eta }_{i}^{({\rm{opt}})}={\tilde{\eta }}_{i}\,\log \,\left(\frac{{\rho }_{i}}{z{\tilde{\eta }}_{i}}\right),$$

and the optimal fatality rate is

20 $${\phi }_{i}^{({\rm{opt}})}=z\frac{{\tilde{\eta }}_{i}}{{\rho }_{i}}.$$

The Lagrange multiplier $z$ is computed by inserting Eq. (19) into Eq. (6) as $z=\exp \,\left[\frac{{\sum }_{i}\,{A}_{i}{\tilde{\eta }}_{i}\,\log \,\left(\frac{{\rho }_{i}}{{\tilde{\eta }}_{i}}\right)-{H}^{({\rm{total}})}}{{\sum }_{i}\,{A}_{i}{\tilde{\eta }}_{i}}\right]\simeq 12.9$. We call the hospital density in Eq. (19) *optimal* in a sense that it minimizes the objective function given in Eq. (18). If the objective function is changed, the optimal density may be changed, which is discussed in the Summary and Discussion section and in the Supplementary Information (SI).

**Figure 4:**
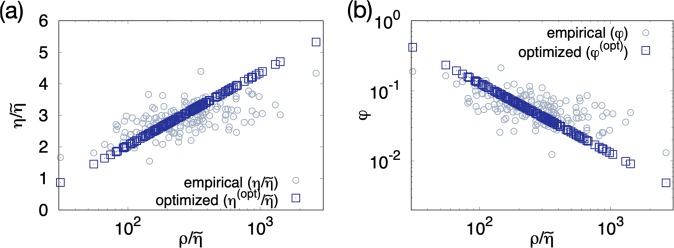
The rescaled hospital density and fatality rate before and after optimization as functions of the rescaled patient density. (**a**) Plots of the rescaled hospital density, $\frac{\eta }{\tilde{\eta }}$ (circle) and $\frac{{\eta }^{({\rm{opt}})}}{\tilde{\eta }}$ (square) versus the rescaled patient density $\frac{\rho }{\tilde{\eta }}$ in semi-logarithmic scale. $\tilde{\eta }$ is the characteristic hospital density estimated empirically as Eq. (16). The data points for the optimized hospital density lie on the line corresponding to Eq. (19) with $z=12.9$. (**b**) Plots of the fatality rate $\phi$ (circle) and ${\phi }^{({\rm{opt}})}$ (square) versus the rescaled patient density $\frac{\rho }{\tilde{\eta }}$. The data points for the optimal fatality rates are on the line corresponding to Eq. (20).

Equations (19) and (20) are the main results of the present study. Remarkably the optimal hospital density and the patient density are rescaled commonly by ${\tilde{\eta }}_{i}$ and then related to each other logarithmically. In Fig. 4(a), the arrangement of the data points of the optimal hospital densities on a straight line is contrasted with the scattered distribution of the current (empirical) hospital densities in the ($\rho /\tilde{\eta },\eta /\tilde{\eta }$) plane in semi-logarithmic scale. The same phenomenon is observed for the fatality rate; the empirical fatality rates ${\phi }_{i}$’s are scattered but the optimized fatality rates lie on a straight line in the ($\rho /\tilde{\eta },\phi$) plane in logarithmic scale as shown in Fig. 4(b). The rescaled patient density ranges between 30.80 (Yeonggwang-gun) and 2646 (Songpa-gu), and is larger than $z\simeq 12.9$ and thus guarantees ${\eta }_{i}^{({\rm{opt}})} > 0$ for all $i$ in Eq. (19). More plots of the optimized hospital densities and fatality rates are given in Fig. S2.

**Figure 5:**
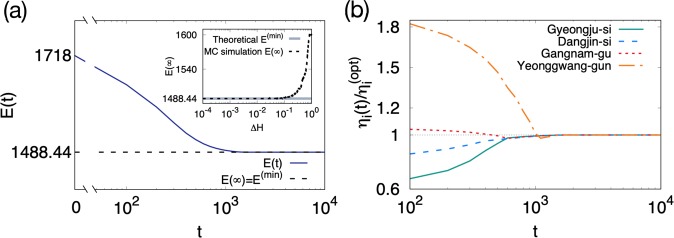
Monte-Carlo (MC) simulation for optimizing the hospital distribution. (**a**) The total energy $E(t)={E}_{{\rm{fatalities}}}$ as a function of the MC step $t$ in the MC simulation with $\Delta H={10}^{-2}$. It becomes stationary at $E(\infty )={E}^{({\rm{\min }})}=1488.44$ for $t\gtrsim {10}^{3}$. Inset: The stationary-state value $E(\infty )$ depends on the increment $\Delta H$. (**b**) The ratio $\frac{{\eta }_{i}(t)}{{\eta }_{i}^{({\rm{opt}})}}$ is plotted as a function of the MC step $t$ for selected districts with $\Delta H={10}^{-2}$. It converges to one for $t\gtrsim {10}^{3}$.

The scattered distributions of the empirical data in Fig. 4 show clearly the deviation of the current distribution of hospitals from the optimum minimizing the total fatalities of TB. The minimized total fatalities ${E}_{{\rm{fatalities}}}^{({\rm{\min }})}$ obtained from the optimal hospital distribution is

21 $${E}_{{\rm{fatalities}}}^{({\rm{\min }})}=\sum _{i}\,{N}_{i}\frac{z\,{\tilde{\eta }}_{i}}{{\rho }_{i}}=z\,\sum _{i}\,{A}_{i}{\tilde{\eta }}_{i}\simeq 1488.44,$$

which is smaller than the current value, 1718, by 13%. For this optimization, ${\sum }_{i}\,{A}_{i}|{\eta }_{i}^{({\rm{opt}})}-{\eta }_{i}|/2\simeq 24.4$ hospitals among a total of ${H}_{{\rm{total}}}=328$ are relocated. In the zero-temperature Monte Carlo (MC) simulation in which some small amount $\Delta H$ of hospitals are moved between randomly selected districts only when the attempted relocation reduces ${E}_{{\rm{fatalities}}}$, the theoretical predictions in Eqs. (19) and (21) are realized in the steady state as long as $\Delta H$ is sufficiently small [Fig. 5(a)]. If ${H}_{i}$’s are restricted to be integers ($\Delta H\,=\,1$), the total fatalities in the steady state is 1599.45. The stationary-state results remain unchanged in the simulations with different initial configurations or with gradually cooling down the temperature. For more details of the simulations, see Methods.

**Figure 6:**
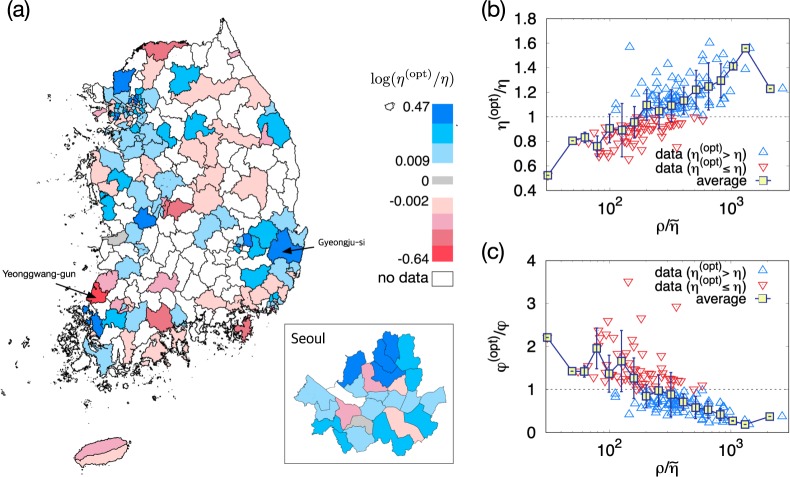
Changes of the hospital density by optimization. (**a**) The logarithmic ratio of the optimal to current hospital density $\log \left(\frac{{\eta }^{({\rm{opt}})}}{\eta }\right)$ is encoded by color for each district of Korea. 85 districts are white, as they do not have both ${\eta }^{({\rm{opt}})}$ and $\eta$ available. (**b**) Plot of the ratio $\frac{{\eta }^{({\rm{opt}})}}{\eta }$ versus the rescaled patient density $\frac{\rho }{\tilde{\eta }}$. Upper and lower triangles are used for the data points with the optimal hospital density larger and smaller, respectively, than the current one. The filled square is the average and the errorbar is the standard deviation of the ratio $\frac{{\eta }^{({\rm{opt}})}}{\eta }$. (**c**) Plot of the ratio of the optimal to current fatality rate $\frac{{\phi }^{({\rm{opt}})}}{\phi }$ versus $\frac{\rho }{\tilde{\eta }}$.

To achieve such reduction in the total fatalities, the hospital density should be increased in some districts and decreased in others [Fig. 5(b)]. For instance, Gyeongju-si should have its hospital density 1.61 times larger than the current hospital density but Yeonggwang-gun 0.534 times larger than the current one. In Fig. 6(a), 143 districts are colored blue (red) if the optimal hospital density is larger (smaller) than the current hospital density. Interestingly, the ratio $\frac{{\eta }^{({\rm{opt}})}}{\eta }$ of the optimal to current hospital density turns out to depend strongly on the rescaled patient density $\frac{\rho }{\tilde{\eta }}$ [Fig. 6(b)]. It implies that if the rescaled patient density is large in a district and small in another district, it is recommended to move some hospitals from the latter to the former district. The high and low values of $\frac{{\eta }^{({\rm{opt}})}}{\eta }$ of Gyeongju-si and Yeonggwang-gun can be understood in this line, as they have quite different values of $\frac{\rho }{\tilde{\eta }}$, 662 and 30.8, respectively. Such a significant correlation is absent between $\frac{{\eta }^{({\rm{opt}})}}{\eta }$ and the raw patient density $\rho$ [Fig. S3]. The change of the fatality rate shows the opposite trend to that of the hospital density; The districts with large (small) rescaled patient density $\frac{\rho }{\tilde{\eta }}$ have their fatality rate decreased (increased) as they gain (lose) hospitals [Fig. 6(c)]. It is remarkable that the changes of the hospital density and the fatality rate are determined not by the patient density or the population density but the rescaled patient density.

## Summary and Discussion

We have here proposed a modeling framework which predicts the optimal distribution under a general objective function, going beyond the previous descriptive explanations for the facility distribution. In deriving the optimal hospital distribution over districts for minimizing the TB fatalities in the whole country, we have found that the characteristic hospital density of each district plays an important role in the optimization. The random-walk nature of the coarse-grained trajectories of patients has been assumed in establishing a theoretical model, and the lattice constant of each district has been introduced to connect the theoretical results and the empirical data. The incorporation of such heterogeneity of districts in the theoretical study of the facility optimization is done only in the present study and can be useful in future studies.

Examining the assumptions and limitations of the proposed model may help better understand and improve its predictive power. The exponential decay of the fatality rate with the hospital density given in Eq. (14) is valid when the dimensionless hospital density is low, $\frac{\sqrt{\lambda \tau }}{\log \,\tau }\ll 1$^26^. The empirical data analyzed in the present study stay in this regime; $\frac{\sqrt{\lambda \tau }}{\log \,\tau }$ ranges between 0.23 and 0.37. If we were to extend to the case of high hospital density or the hospital locations being no more independent of one another, the fatality rate might behave differently from Eq. (14). For ${\phi }_{i}=f({\eta }_{i}/{\tilde{\eta }}_{i})$ with $f(x)$ a decreasing convex function such as exponential, stretched exponential, or power law, the optimal hospital density will be given by $\frac{{\eta }_{i}}{{\tilde{\eta }}_{i}}={(-f{\prime} )}^{-1}\left(z\frac{{\tilde{\eta }}_{i}}{{\rho }_{i}}\right)$ with ${(f{\prime} )}^{-1}$ being the inverse of the derivative of $f(x)$. The relation between the two rescaled variables $\frac{{\eta }_{i}}{{\tilde{\eta }}_{i}}$ and $\frac{{\rho }_{i}}{{\tilde{\eta }}_{i}}$ depends on the specific form of $f(x)$ and reduces to Eq. (19) in case of $f(x)={e}^{-x}$. The case of $f(x)$ being a power law is presented in the SI. Regarding the robustness of the functional form of the fatality rate, it will be of interest to investigate which model of walk with traps exhibits such a power-law survival probability.

We have counted only the private general hospitals, but there are mostly found one or two public health centers in a district, which can also provide the medical treatment to TB patients although its portion may not be large^35^. One can optimize the private hospital distribution considering the contribution of the public health centers to the TB treatment, which is presented in the SI and Fig. S4. The results remain unchanged qualitatively. Extending the trajectories of patients to the nearby districts can be one way of making the model more realistic, which will address how the similarity or dissimilarity of adjacent districts may affect the fatality rate and the total fatalities. A correlation may be expected between the economic level of a district and the fatality rate of a disease, which is not significant in case of TB in Korea [Fig. S5], as the TB treatment is covered by the national medical insurance in Korea^36^, but should be considered in the application to other diseases or other countries. When a given number of hospitals can be opened or should be shut down for financial or other reasons, our results will be helpful for the investigation of the optimal locations.

## Methods

### Data-sets

Under the control of the Ministry of Health and Welfare, several organizations such as the Korean National Tuberculosis Association and Korea Centers for Disease Control and Prevention cooperate to prevent and eradicate tuberculosis in Korea^36^. The related information has been well recorded, which is accessible through Statistics Korea^20^. The data-sets used in the present study have been collected district by district. As a result, 37347 new TB patients, 330 hospitals, and 2127 dead patients for 228 districts in year 2014 have been considered in the present study. The hospitals considered in our study are the private ones classified as general or superior general hospitals in the Korean Medical Service Act. The theoretical modeling for the fatality rate applies for ${I}_{s}=143$ districts which have at least one hospital and at least one dead patient. The total number of new and dead patients, and hospitals in those 143 districts are 32322, 1718, and 328.

### Monte carlo simulation

To illustrate the hospital relocation process, we perform the zero-temperature Monte Carlo (MC) simulation in which hospitals are relocated over ${I}_{s}$ districts towards decreasing the energy, equal to the total fatalities given in Eq. (21), as follows:

(i) Initially the number of hospitals in each district is set equal to the empirical data.
(ii) For two randomly selected districts $i$ and $j$, consider moving $\Delta H$ hospitals from $i$ to $j$ as long as ${H}_{i}-\Delta H > 0$.
(iii) Accept this relocation if the energy change $\Delta E={N}_{i}({e}^{-\frac{{H}_{i}-\Delta H}{{A}_{i}{\tilde{\eta }}_{i}}}-{e}^{-\frac{{H}_{i}}{{A}_{i}{\tilde{\eta }}_{i}}})+{N}_{j}({e}^{-\frac{{H}_{j}+\Delta H}{{A}_{j}{\tilde{\eta }}_{j}}}-{e}^{-\frac{{H}_{j}}{{A}_{j}{\tilde{\eta }}_{j}}})$ is zero or negative. Reject it otherwise.
(iv) Repeat steps (ii) and (iii) ${I}_{s}$ times to increase the MC step $t$ by one.

We find that the energy becomes stationary around $t={10}^{3}$ MC steps and thus we run the simulations just up to 10^4^ MC steps [Fig. 5(b)]. $\Delta H$ represents the amount of hospitals moved by one relocation. For $\Delta H\lesssim 0.05$, the hospital configuration and the energy in the stationary state coincide with the theoretical predictions in Eqs. (19) and (21), respectively. Replacing the initial hospital configuration by a random one, the energy and the hospital configuration in the stationary-state are not changed but remain the same as the theoretical prediction. Since a hospital relocation is accepted only when the corresponding energy change is not positive, this simulation corresponds to zero temperature $T=0$. We have also run the MC simulation with lowering temperature gradually from $T=20$ to $T=3\times {10}^{-9}$ but the hospital configuration and the energy in the stationary state are found to be the same as those of the zero-temperature MC simulation.

## Supplementary information

Supplementary Information.

## Data Availability

The datasets generated during the current study are available from the corresponding author on reasonable request.
