# Supplementary material for: Optimizing hospital distribution across districts to reduce tuberculosis fatalities
Source: Sci Rep. 2020 May 25;10:8603. doi: 10.1038/s41598-020-65337-x (PMC7248084; doi:10.1038/s41598-020-65337-x)
Supplement: Supplementary file 1 — Supplementary Information. [file 41598_2020_65337_MOESM1_ESM.pdf]

Supplementary Information: Optimizing hospital distribution across districts to reduce tuberculosis fatalities

Mi Jin Lee[1], Kanghun Kim[2], Junik Son[3], and Deok-Sun Lee[1,*]

[1]Department of Physics, Inha University, Incheon 22212, Korea
[2]Financial Engineering Team, Meritz Securities, Seoul 07326, Korea
[3]Department of Family Medicine, Daejeon Sun Medical Center, Daejeon 34811, Korea

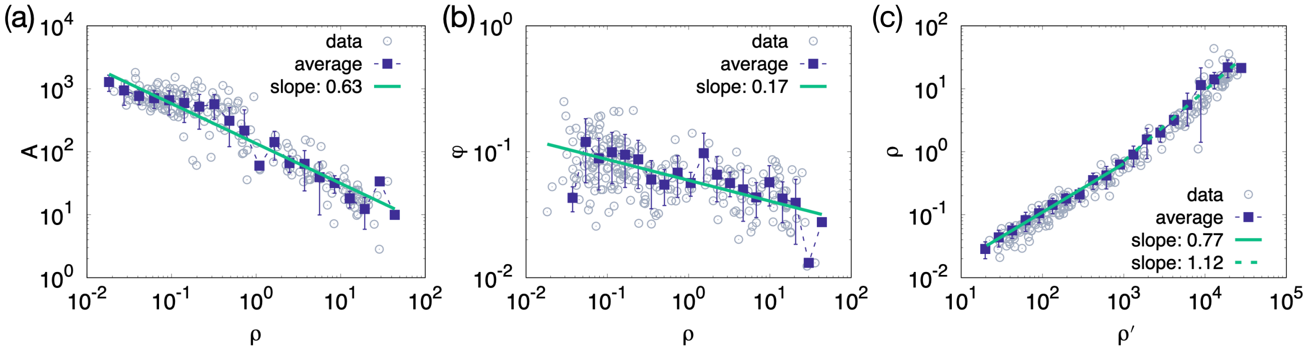

Figure S1. Scaling behaviors of the properties of districts. (**a**) Plot of the area A versus the patient density ρ. (**b**) Plot of the fatality rate ϕ versus the patient density ρ. (**c**) Plot of the patient density ρ and the whole population density ρ'. In all the panels, the empirical data points (open circles) and the average values (filled squares) as functions of ρ or ρ' are presented. The errorbar is the standard deviation. In the panel (**c**), the two solid lines having slopes 0.77 and 1.12 fit the average values in the range $\rho' \leq 10^3$ and $\rho' > 10^3$, respectively.

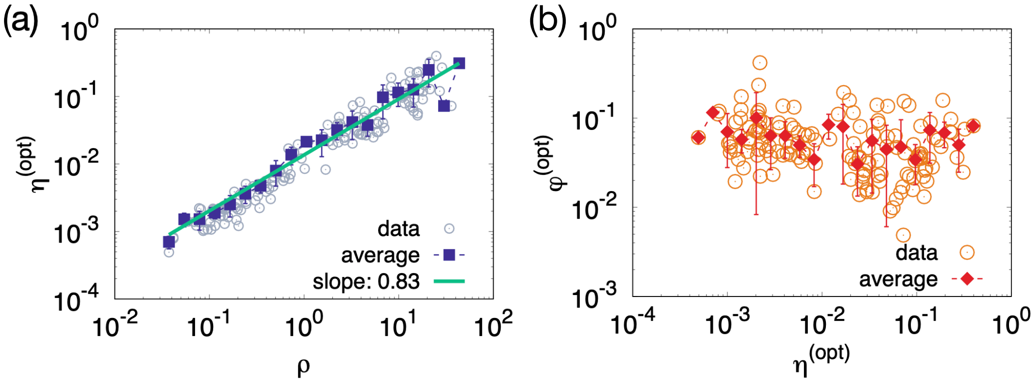

Figure S2. Behaviors of the optimized hospital density and fatality rates. (**a**) Plot of the optimal hospital density $\eta^{(\mathrm{opt})}$ versus the patient density ρ. The filled square is the average and the errorbar is the standard deviation. The solid line fits the average of $\eta^{(\mathrm{opt})}$ as a function of ρ. (**b**) Plot of the optimal fatality rate $\phi^{(\mathrm{opt})}$ versus the optimal hospital density $\eta^{(\mathrm{opt})}$. The filled diamond is the average and the errorbar is the standard deviation.

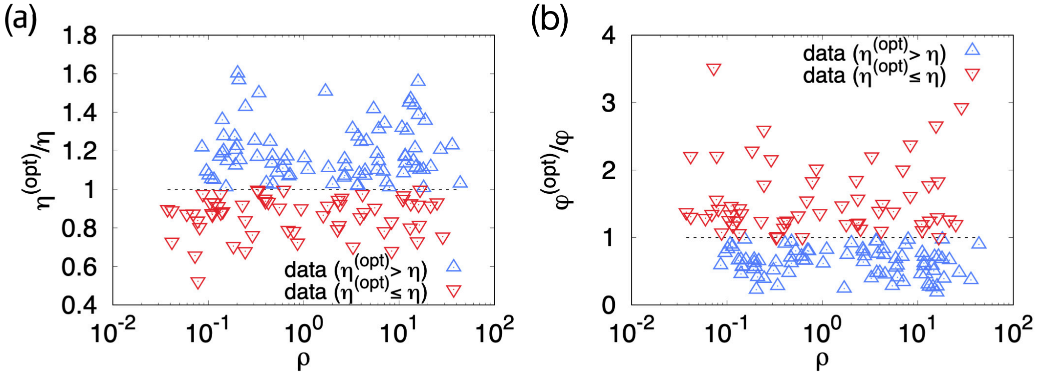

Figure S3. The relation between the changes made by optimization and the raw patient density. (**a**) Plot of the ratio of the optimal to current hospital density $\frac{\eta^{(\mathrm{opt})}}{\eta}$ versus the raw patient density ρ. The data points are blue (red) for $\eta^{(\mathrm{opt})} > \eta$ (for $\eta^{(\mathrm{opt})} < \eta$). The data points are scattered, showing no significant correlation. (**b**) Plot of the ratio of the optimal to current fatality rate $\frac{\phi^{(\mathrm{opt})}}{\phi}$ versus the raw patient density ρ. No correlation is seen.

Optimizing hospital density under the fatality rate in a power-law form

Here we investigate the optimal hospital density in case of $\phi(\eta)$ given as a power law. Suppose that the fatality rate $\phi_i(\eta_i)$ takes the form

$$\phi_i(\eta_i) = f\left(\frac{\eta_i}{\tilde{\eta}_i}\right), \tag{S1}$$

with $\tilde{\eta}_i$ the characteristics hospital density of district i and $f(0) = 1$. Then the total fatalities in Eq. (7) with ϕ_i in Eq. (S1) is minimized when $\delta E = \sum_i A_i \delta\eta_i \left(\rho_i \frac{\partial \phi_i}{\partial \eta_i} + z\right) = 0$ is satisfied or

$$-f'\left(\frac{\eta_i}{\tilde{\eta}_i}\right) = z\frac{\tilde{\eta}_i}{\rho_i}. \tag{S2}$$

To be specific, let us consider the fatality rate given in the following power-law form:

$$\phi_i = \left(1 + \frac{\eta_i}{\tilde{\eta}_i}\right)^{-\gamma} \tag{S3}$$

with γ a constant. By Eq. (S2), the optimal hospital density is determined as $\gamma\left(1 + \frac{\eta_i^{(\mathrm{opt})}}{\tilde{\eta}_i}\right)^{-\gamma-1} = z\frac{\tilde{\eta}_i}{\rho_i}$, leading to

$$\frac{\eta_i^{(\mathrm{opt})}}{\tilde{\eta}_i} = \left(\frac{\gamma\rho_i}{z\tilde{\eta}_i}\right)^{\frac{1}{\gamma+1}} - 1. \tag{S4}$$

The Lagrange multiplier z is determined by the constraint in Eq. (6) and evaluated in this case as

$$z^{\frac{1}{\gamma+1}} = \frac{\sum_i A_i \tilde{\eta}_i \left(\frac{\gamma\rho_i}{\tilde{\eta}_i}\right)^{\frac{1}{\gamma+1}}}{H^{(\mathrm{total})} + \sum_i A_i \tilde{\eta}_i}, \tag{S5}$$

and the optimal fatality rate is

$$\phi_i^{(\mathrm{opt})} = \left(\frac{z\tilde{\eta}_i}{\gamma\rho_i}\right)^{\frac{\gamma}{\gamma+1}}. \tag{S6}$$

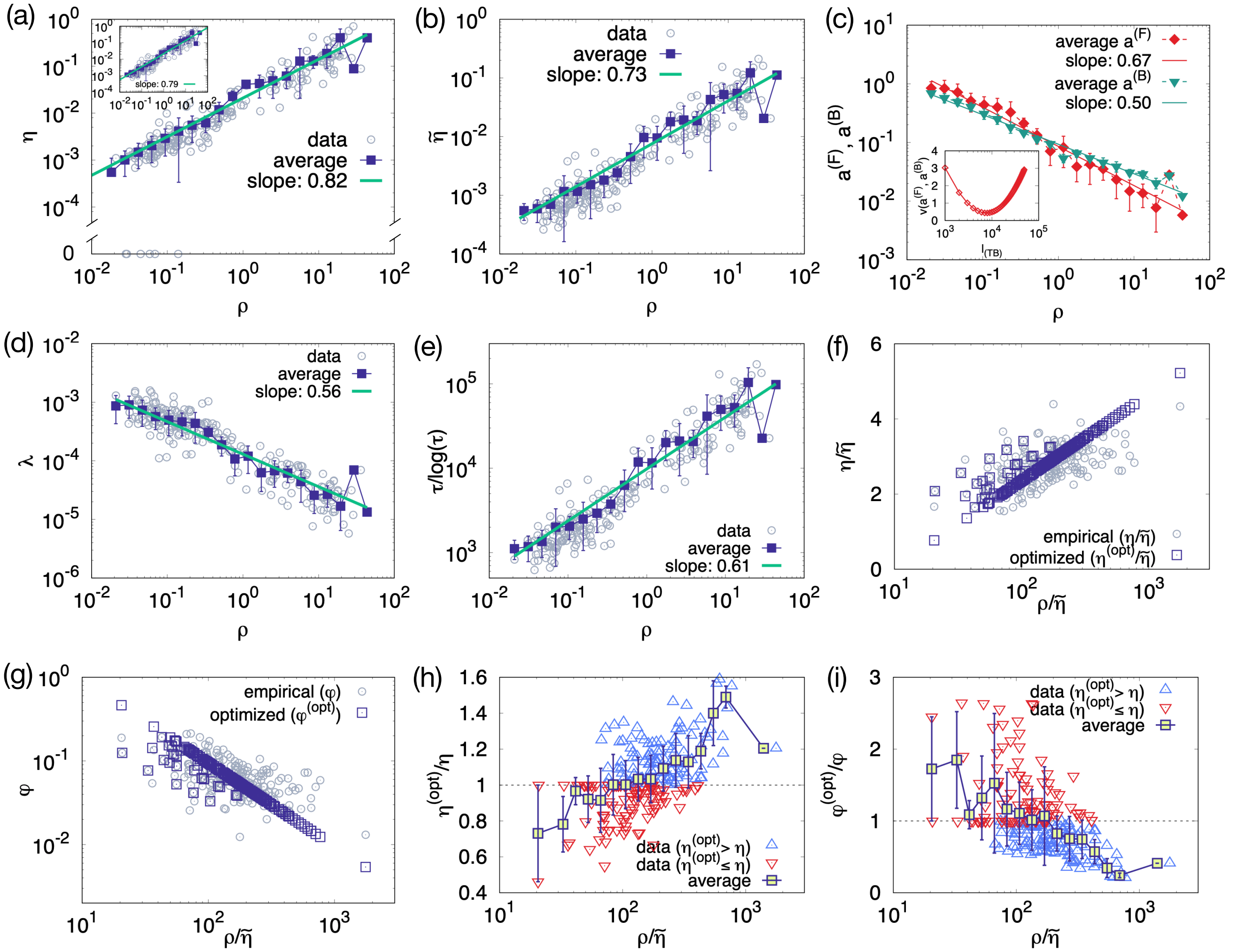

Figure S4. Results of the optimization including the public health centers. (**a**) Plot of the integrated hospital density $\eta = \eta^{(\text{private})} + \eta^{(\text{public})}$ versus the patient density ρ. Inset: the same plot for the districts having nonzero η and nonzero ρ. (**b**) Plot of the integrated characteristic hospital density $\tilde{\eta}$ versus the patient density ρ. (**c**) Plot of the lattice constant $a^{(\text{F})}$, obtained by using the integrated characteristic density in Eq. (15) with $\ell_{(\text{TB})}{}^{*} = 8000$ km, versus the patient density ρ, compared with $a^{(\text{B})}$. Inset: The logarithmic distance between $a^{(\text{F})}(\ell_{(\text{TB})})$ and $a^{(\text{B})}$ as a function of $\ell_{(\text{TB})}$. It is the minimum at $\ell_{(\text{TB})} = 8000$ km. (**d**) Plots of the dimensionless hospital density λ versus the patient density ρ. (**e**) Plots of $\tau/\log\tau$ versus the patient density ρ. (**f**) Plots of the rescaled hospital density $\frac{\eta}{\tilde{\eta}}$ before and after optimization versus the rescaled patient density $\frac{\rho}{\tilde{\eta}}$. 33 districts have $\eta_i^{(\text{opt})} = \eta_i^{(\text{public})}$, so their optimized data points deviate from the straight line. (**g**) Plots of the fatality rate ϕ before and after optimization versus the rescaled patient density $\frac{\rho}{\tilde{\eta}}$. (**h**) Plots of the ratio of the hospital density before and after optimization $\frac{\eta^{(\text{opt})}}{\eta}$ versus the rescaled patient density $\frac{\rho}{\tilde{\eta}}$. (**i**) Plots of the ratio of the fatality rate before and after optimization $\frac{\phi^{(\text{opt})}}{\phi}$ versus the rescaled patient density $\frac{\rho}{\tilde{\eta}}$.

Analysis including the public health centers

Most districts have one or two public health centers each. They are also in charge of providing the medical treatment for TB patients. Therefore it may be interesting to incorporate the contribution of public health centers to the fatality rate in the investigation of the optimal distribution of private hospitals.

Let the hospital density of a district be the sum of public and private ones as

$$\eta_i = \eta_i^{(\text{private})} + \eta_i^{(\text{public})} \geq \eta_i^{(\text{public})}, \tag{S7}$$

where $\eta_i^{(\text{private})}$ is equal to the hospital density considered in the main text, defined as the ratio of the number of private hospitals to the area of district i, and $\eta_i^{(\text{public})}$ is the ratio of the number of public health centers to the district's area. The characteristic hospital density $\tilde{\eta}_i$ is computed by using Eq. (S7) in Eq. (16). These integrated hospital density and characteristic density are plotted as functions of the patient density in Figs. S4(a) and S4(b), respectively. The scaling exponents are similar to those obtained when only the private hospitals are considered.

The lattice constant $a^{(\text{F})}$ obtained by using the integrated characteristic density in Eq. (15) with $\ell_{(\text{TB})}{}^* = 8000$ km and $a^{(\text{B})}$ are compared as functions of the patient density in Fig. S4(c), and the dimensionless hospital density λ and the number of steps τ of each district are given in Fig. S4(d) and S4(e), respectively. The reasonable agreement of $a^{(\text{F})}$ and $a^{(\text{B})}$, the decrease of λ and the increase of $\tau/\log\tau$ with increasing the patient density ρ are observed as in the case of considering the private hospitals only.

Let us consider the relocation of private hospitals, fixing public health centers, across districts. The hospital density should be equal to or larger than the fixed public health center density, i.e., $\eta_i \geq \eta_i^{(\text{public})}$. Note that the constraint $\eta_i > 0$ is used in the main text where only the private hospitals are considered. For the relocation of private hospitals, we consider 217 districts which have at least one private or public hospital and non-zero fatality rate. The total number of hospitals in those districts is 568, and the total fatalities is 2108 in the empirical data.

Incorporating the inequality constraint of Eq. (S7) as well as the equality of Eq. (6) in the optimization, we find the Karush-Kuhn-Tucker (KKT) conditions[1–3] in minimizing the total fatalities as

$$\frac{\delta}{\delta \eta_i} \sum_i \left[N_i \exp\left(-\frac{\eta_i}{\tilde{\eta}_i}\right) - z\left(H^{(\text{total})} - \sum_i \eta_i A_i\right) - w_i\left(\eta_i - \eta_i^{(\text{public})}\right)\right] = 0, \tag{S8}$$

with z and w_i's called the Lagrange multiplier and the KKT multipliers respectively. Then the optimal hospital density, including both public and private, is given by

$$\eta_i^{(\text{opt})}(z, w_i) = \tilde{\eta}_i \log\left(\frac{1}{z + \frac{w_i}{A_i}} \frac{\rho_i}{\tilde{\eta}_i}\right), \tag{S9}$$

and the optimal fatality rate is

$$\phi_i^{(\text{opt})}(z, w_i) = \left(z + \frac{w_i}{A_i}\right) \frac{\tilde{\eta}_i}{\rho_i}. \tag{S10}$$

These are reduced to Eqs. (19) and (20), respectively, if $w_i = 0$. To meet the inequality and equality conditions, it is known[1–3] that the optimal hospital density either satisfies $w_i = 0$ or $\eta_i = \eta_i^{(\text{public})}$ for every i. Therefore the true optimal solution $\phi^{(\text{opt})}(z^{(\text{opt})}, w_i^{(\text{opt})})$ can be found practically by finding $z^{(\text{opt})}$ with which i) the optimal hospital density of every district is given either by Eq. (S9) with $w_i = 0$ or by $\eta_i^{(\text{opt})} = \eta_i^{(\text{public})}$, and ii) the total number of hospitals is equal to the empirical value as in Eq. (6). To determine $z^{(\text{opt})}$ and $\{w_i^{(\text{opt})}\}$, we use the following algorithm:

(i) For given z, the optimal hospital density $\eta_i^{(\text{opt})}(z)$ is determined as follows. First use $w_i = 0$ in Eq. (S9) to obtain $\eta_i^{(\text{opt})}(w_i = 0, z)$ for every district i. If it is equal to or larger than the public center density $\eta_i^{(\text{public})}$, then accept it as $\eta_i^{(\text{opt})}(z)$. Otherwise, $\eta_i^{(\text{opt})}(z)$ is set equal to $\eta_i^{(\text{public})}$, leaving a negative value of $w_i^{(\text{opt})}$ by Eq. (S9). In summary, $\eta_i^{(\text{opt})}(z) = \max\{\eta_i^{(\text{opt})}(w_i = 0, z), \eta_i^{(\text{public})}\}$. $w_i^{(\text{opt})} = 0$ if the former is chosen and $w_i^{(\text{opt})} = A_i[e^{-\frac{\eta_i^{(\text{public})}}{\tilde{\eta}_i}} \frac{\rho_i}{\tilde{\eta}_i} - z]$ otherwise.

(ii) After running step (i) for all districts, compute the predicted total number of hospitals $H_{\text{total}}^{(\text{opt})}(z) = \sum_i A_i \eta_i^{(\text{opt})}(z)$

(iii) Repeat steps (i) and (ii) for z between 0 and 30 with increment 0.0001.

(iv) Determine $z^{(\mathrm{opt})}$ with which the predicted total number of hospitals is the closest to the empirical value, i.e., $|H^{(\mathrm{opt})}_{\mathrm{total}}(z) - H_{\mathrm{total}}|$ is minimized.

We find that the predicted total number of hospitals is closest to the empirical value 568 at $z^{(\mathrm{opt})} = 9.5175$, with which $H^{(\mathrm{opt})}_{\mathrm{total}}(z^{(\mathrm{opt})}) = 567.9996$, and 33 districts have only public health centers with no private hospital, i.e., $\eta_i^{(\mathrm{opt})} = \eta_i^{(\mathrm{public})}$. The total fatalities is reduced to 1878.48, smaller than the current value 2108 by 11%.

The scattered distribution of the empirical data and the line alignment of the optimized hospital density in the $(\rho/\tilde{\eta}, \eta/\tilde{\eta})$ plane are also shown in Fig. S4(f). Some points for the optimal hospital density deviate from the aligned line, which are from the 33 districts having $\eta_i^{(\mathrm{opt})} = \eta_i^{(\mathrm{public})}$ in the optimized state. The scaling relation between the optimized fatality rate and the rescaled patient density predicted by Eq. (S10) is also shown in Fig. S4(g) with the same kind of deviations as in Fig. S4(f). The changes of the hospital density and of the fatality rate by the optimization are correlated with the rescaled patient density positively and negatively as shown in Figs. S4(h) and S4(i), respectively. These correlations are identical to those in the case of considering the private hospitals only. All these results suggest that even when including the public health centers, all the analysis results remain the same qualitatively.

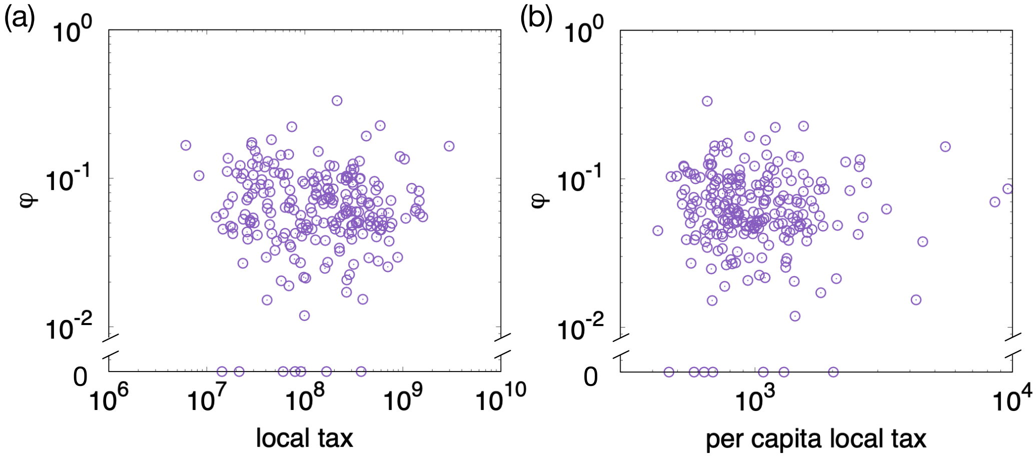

Figure S5. The relation between the local tax and the fatality rate. The local tax is considered here as a measure of the economic level of a district. (**a**) Plot of the fatality rate of TB versus the local tax in logarithmic scales. The Pearson correlation coefficient is 0.031 and the P value is 0.64. (**b**) Plot of the fatality rate of TB versus the per capita local tax in logarithmic scales. The Pearson correlation coefficient is 0.015 and the P value is 0.82.

References

1. Kuhn, H. W. & Tucker, A. W. Nonlinear programming. In *Proceedings of the Second Berkeley Symposium on Mathematical Statistics and Probability*, 481–492 (University of California Press, Berkeley, Calif., 1951).
2. Karush, W. Minima of functions of several variables with inequalities as side conditions (2014).
3. Kjeldsen, T. H. A contextualized historical analysis of the Kuhn-Tucker theorem in nonlinear programming: the impact of World War II. *Hist. Math.* **27**, 331–361 (2000).
